# Constitutive Model of Quasi-Static and Dynamic Tensile Behavior and High-Temperature Rheology of PEEK

**DOI:** 10.3390/ma18225127

**Published:** 2025-11-11

**Authors:** Lizhi Tian, Jiaxin Deng, Xin Zhang, Bing Wang, Tiegang Tang, Lei Lu, Cheng Fan, Chun Zhang

**Affiliations:** 1 Institute of Fluid Physics, China Academy of Engineering Physics, Mianyang 621999, China; tianlz20@caep.cn (L.T.);; 2 School of Aeronautics, Northwestern Polytechnical University, Xi’an 710072, China; 3 Fujian Provincial Key Laboratory of Terahertz Functional Devices and Intelligent Sensing, School of Mechanical Engineering and Automation, Fuzhou University, Fuzhou 350108, China; 4 Extreme Material Dynamics Technology Laboratory, Chengdu 610000, China

**Keywords:** polyether-ether-ketone, constitutive model, temperature dependence, strain rate dependence

## Abstract

Static and dynamic uniaxial tensile responses were investigated to accurately characterize and predict the mechanical properties of PEEK (polyether-ether-ketone) at strain rates ranging from 10^−3^ s^−1^ to 200 s^−1^ and temperatures ranging from 23 °C to 110 °C. The tensile responses showed dependences on the strain rate and temperature, and the dependences of the yield strength and elastic modulus on the temperature and strain rate were studied. A modified phenomenological Sherwood–Frost constitutive model considering a wide range of strain rates and temperatures was established to characterize the tensile mechanical response of PEEK material before yielding based on the experimental data. The results indicate that the model can accurately describe the pre-yield behavior of PEEK under different temperature and strain rate conditions, thus reducing the dependency on experimental data for subsequent researchers, thereby providing a theoretical foundation and modeling framework for the design and performance evaluation of CF/PEEK composite structures.

## 1. Introduction

Carbon fiber-reinforced polyether-ether-ketone composite (CF/PEEK) is a high-performance composite comprising polyether-ether-ketone (PEEK) as the matrix and carbon fiber (CF) as the reinforcing phase. The exceptional toughness of PEEK (elongation at break > 50%) and its thermal stability (glass transition temperature ~143 °C) position CF/PEEK as an ideal material for lightweight blast-resistant applications, such as counter-terrorism protective equipment and hazardous chemical transport containers [1,2,3,4]. The strong plastic deformation capability of PEEK enables efficient impact energy dissipation, effectively suppressing crack propagation. However, under impact loading, CF/PEEK composites experience progressive failure mechanisms including matrix cracking, interfacial debonding, delamination, and cascading damage. Although matrix cracking does not directly induce structural failure, it triggers interfacial damage—a critical factor for performance degradation. Simultaneously, in service scenarios involving external high-temperature environments or explosive combustion-to-deflagration transitions, CF/PEEK composite structures may undergo nearly 100 °C thermal pre-loading before sustaining explosive impacts. The thermal softening effect on PEEK under such conditions exacerbates interfacial damage [5,6], leading to a significant degradation of interlaminar performance and posing severe safety risks to blast-resistant composite structures. To accurately predict CF/PEEK’s mechanical response under coupling conditions and ensure structural integrity, systematic characterization of the PEEK matrix’s mechanical behavior across a broad temperature range (20 °C to 110 °C) and a wide strain rate spectrum (10^−3^ s^−1^ to 10^3^ s^−1^) is essential. Such data will provide critical theoretical foundations for optimizing protective structure design and performance evaluation.

Currently, extensive research on PEEK’s mechanical properties has yielded various constitutive models for predicting its behavior under complex loading. In terms of fundamental mechanical properties, Chen et al. [7,8] studied PEEK and its composites under uniaxial tensile tests of various strain rates. The results demonstrated that PEEK exhibits relatively low sensitivity to low-to-moderate strain rates in ultimate stress but shows significant strain rate dependence under high strain rates, while failure strain decreases markedly with increasing strain rates. This strain rate dependency is attributed to enhanced interfacial toughness at elevated strain rates, which delays crack propagation through energy dissipation mechanisms. Tang et al. [9] systematically investigated the mechanical behavior and failure mechanisms of PEEK and its composites under a comprehensive range of temperatures (−30 °C to 110 °C) and strain rates (10^−3^ s^−1^ to 10^3^ s^−1^). The study revealed that tensile strength exhibits strong correlations with both strain rate and temperature. Notably, at extreme temperatures (high/low), the failure strain of PEEK composites becomes less sensitive to strain rate variations, accompanied by reduced energy absorption capacity. This phenomenon is linked to thermal–mechanical coupling effects that alter polymer chain mobility and interfacial adhesion under thermal softening conditions.

To characterize the temperature and strain rate sensitivity of PEEK, researchers have developed two categories of constitutive models:

1. Physically based constitutive models

These models are constructed based on microscopic physical mechanisms during material deformation. A representative model is the Mulliken–Boyce model [10], which incorporates chain segment motion, Ree–Eyring activation volumes, and micro-relaxation processes. While this model can accurately predict the mechanical behavior of thermoplastic materials, its complex mathematical formulations, numerous parameters, and high sensitivity limit its practical applicability under broad temperature–rate ranges.

2. Phenomenological constitutive models

Developed through experimental data fitting, these models prioritize empirical correlations over mechanistic details. They mainly include product-type models, emphasizing stress decomposition and multiplicative interactions, and viscous models, introducing rate-dependent damping terms. These models are widely used in engineering due to their simplified formulations enabling rapid predictions.

Viscous models decompose stress into instantaneous elasticity, viscoelasticity, and viscoplasticity components, typically modeled via spring–dashpot elements. Chen et al. [11] investigated PEEK’s mechanical behavior using a linear four-element Burgers mechanical model, demonstrating that PEEK resin conforms to a four-parameter Bergstrom model, with model predictions closely aligning with experimental data. El-Qoubaa et al. [12] introduced an apparent activation volume attenuation function within the Ree–Eyring framework, enabling accurate description of PEEK’s mechanical response across ambient temperatures and wide strain rate ranges. Lu et al. [13] presented an anisotropic visco-hyperelastic constitutive model to describe the strain rate–temperature-dependent deformational behavior of CF/PEEK during hot stamping forming. This constitutive model accurately describes the material flow characteristics, strain and stress distributions, and forming defects in CF/PEEK curved beams. Garcia-Gonzalez et al. [14] further developed a hyperelastic–thermoviscoplastic constitutive model that couples temperature and strain rate effects, successfully reproducing PEEK’s complex mechanical behavior. Although viscous constitutive models incorporate rate, temperature, and history-dependent effects within a unified framework, and enable simultaneous prediction of impact, creep, and stress relaxation responses, they generally face challenges including multiple parameters, challenging calibration, high numerical stiffness, and significant extrapolation risks.

Product-type constitutive models primarily include the Johnson–Cook (JC) model, Nasraoui model, Duan–Saigal–Greif–Zimmerman (DSGZ) model, and Sherwood–Frost model:

1. Chen et al. [15,16] investigated PEEK’s tensile deformation and fracture behavior under strain rate and temperature variations. They proposed a modified JC model incorporating strain rate and temperature dependencies, demonstrating superior capability in predicting rheological behavior under high-temperature conditions. JC-based models are typically used to predict post-yield flow stress (plastic deformation phase). However, they cannot describe elastic deformation or other deformation stages (e.g., elastic regimes), requiring supplementary models or parameters for comprehensive characterization.
2. Nasraoui et al. [17] introduced an “additive–multiplicative” hybrid function based on the G’sell–Jonas framework, utilizing dual exponential terms to accurately capture the yield softening and hardening stages of plastic flow. While improving post-yield deformation predictions, this model requires an additional spring element for elastic regime description and lacks validation under high-temperature conditions.
3. The DSGZ model [18] integrates multiple phenomenological frameworks (e.g., the Johnson–Cook, G’Sell–Jonas, Matsuoka, and Brooks models). Zheng et al. [19] applied the DSGZ model to simulate PEEK’s mechanical behavior under high temperatures (293–543 K) and low strain rates (8.3 × 10^−3^–3.3 × 10^−1^ s^−1^). While effective in describing the elastic regime and large-strain responses after hardening, it shows limited predictive accuracy for the nonlinear transitional regime. Zhu et al. [20] further developed a generalized DSGZ model to achieve precise predictions of PEEK’s mechanical properties under quasi-static loading and broad temperature ranges, though validation under moderate-to-high strain rates remains insufficient.
4. Sherwood–Frost [21,22] employs a power-law function to decouple strain, strain rate, and temperature effects, enabling accurate prediction of the impact compression response of polyurethane foam under thermal–impact coupling. However, the strain rate term in this model adopts simplistic logarithmic or power-law formulations that neglect thermal activation saturation effects. This omission leads to persistent overestimation of hardening effects in the high-strain-rate regime, where prediction errors exhibit pronounced amplification as both the temperature and strain rate increase.

Despite progress in PEEK mechanical modeling, critical challenges remain under high-temperature dynamic loading:

1. Inadequate pre-yield behavior characterization: Current models (e.g., JC, DSGZ) predominantly address post-yield plasticity, overlooking the critical linear-elastic-to-nonlinear transition (occurring before fiber fracture at <2% strain). This transitional regime is pivotal for optimizing impact energy absorption in blast-resistant designs.
2. Severe model fragmentation: Most constitutive models only address low- or high-strain-rate behavior in isolation or are limited to narrow temperature ranges. There is a lack of systematic research under coupled conditions of broad temperature (20 °C to 110 °C) and strain rate (10^−3^–10^3^ s^−1^) spectra.

To address these gaps, this study systematically investigates the strain rate and temperature sensitivity of PEEK’s mechanical behavior through quasi-static and dynamic tensile tests. The tests cover a wide range of strain rates and temperatures to comprehensively characterize the material’s viscoelastic–viscoplastic response. Based on the experimental results, a modified phenomenological constitutive model incorporating both temperature and strain rate effects is developed. The results indicate that the model can accurately describe the pre-yield behavior of PEEK under different temperature and strain rate conditions, thus reducing the dependency on experimental data for subsequent researchers, thereby providing a theoretical foundation and modeling framework for the design and performance evaluation of CF/PEEK composite structures.

## 2. Materials and Methods

According to Standard GB/T1040 [23], the quasi-static specimens and the dynamic tensile specimens were prepared by the injection molding method. The raw material employed in this study was polyether-ether-ketone (PEEK) polymer, specifically grade ZYPEEK^®^-330G, manufactured by Ningbo Shuxiang Aerospace Composite Material Co., Ltd. (Ningbo, China). The PEEK injection mold was first mounted on the injection molding machine. Subsequently, PEEK pellets were placed in a vacuum oven and dried at 110 °C for 6 h to remove moisture, thereby preventing potential degradation of mechanical properties during subsequent molding. Following this, the processed pellets were fed into the machine’s hopper. The processing parameters were set as mold temperature 190 °C (above PEEK’s glass transition temperature of 143 °C), nozzle temperature 385 °C, heating zone temperature 380 °C, screw rotation speed 50%, and back pressure 3 MPa to mold the pellets into two standard specimens. After completion of the injection, the mold was opened and the specimens were removed for natural cooling to room temperature. Upon cooling, the specimens were trimmed to remove imperfections, such as flash, in order to prevent potential issues including fixture slippage during testing that could compromise the test results and mechanical properties.

**Figure 1:**
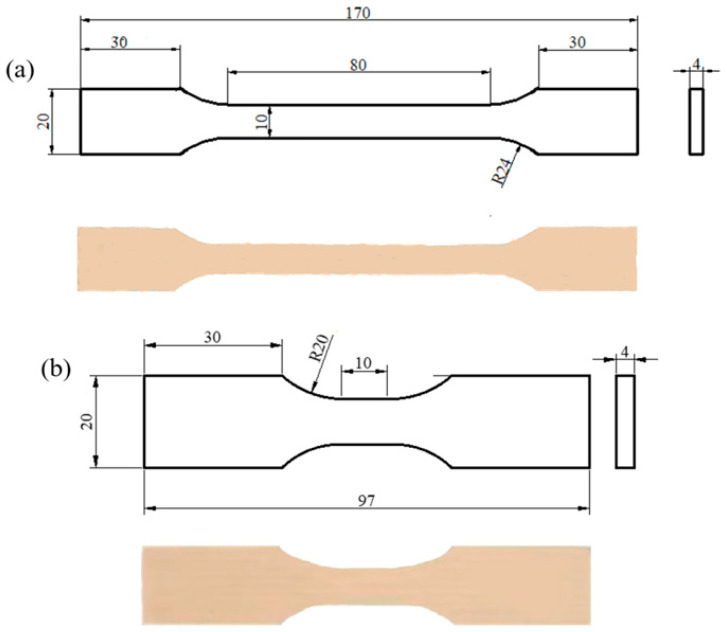
Standard test pieces: (**a**) quasi-static test specimen; (**b**) dynamic test specimen.

The quasi-static test specimens measured 170 mm in length, 20 mm in width, and 4 mm in thickness, while the dynamic test specimens measured 97 mm in length, 20 mm in width, and 4 mm in thickness. The quasi-static specimens’ dimensions are shown in Figure 1a, featuring a standardized dog-bone shape. The dynamic tensile specimens’ dimensions are shown in Figure 1b. These specimens possess a reduced section and appropriate end tabs. Both specimen types were machined to precise tolerances to adhere to normative testing requirements and ensure reproducibility in mechanical characterization. The test conditions for the uniaxial tensile tests are summed up in Table 1.

**Figure 2:**
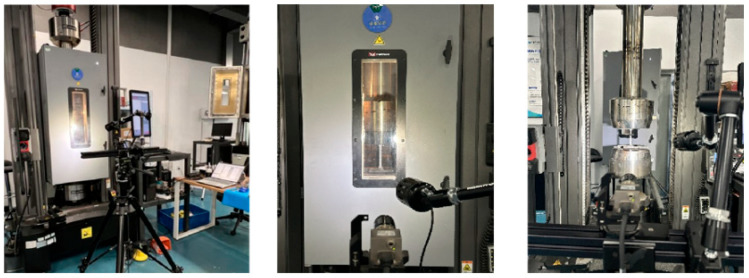
Equipment: Instron 5985 250KN low-/medium-temperature materials testing machine and Instron high-speed testing machine.

Quasi-static tensile tests were conducted on the Instron 5985 250kN low-/medium-temperature materials testing machine (Instron, Norwood, MA, USA), shown in Figure 2. Strain measurements at quasi-static rates were monitored using Digital Image Correlation (DIC), while intermediate-/high-strain-rate tests were performed using the Instron high-speed testing machine (non-Hopkinson bar). Unlike the split Hopkinson pressure bar apparatus, this system utilizes integrated load cells and high-speed data acquisition suited for direct tension tests. This optical technique can map full-field displacement and strain, which particularly emphasizes the elastic modulus, Poisson’s ratio, yield strength, and post-yield behavior. To ensure measurement accuracy, a high-contrast, random speckle pattern was applied uniformly to the gauge region of each specimen using aerosol spray painting. When employing the Digital Image Correlation (DIC) technique, it is important to ensure a uniformly applied speckle pattern on the specimen surface to prevent adverse effects on experimental results. The specimens were loaded at a controlled displacement rate until fracture occurred. With reference to Standard GB/T9979 [24], for elevated temperature testing, the environmental chamber was first heated to the predetermined temperature and maintained for 15 min prior to loading to ensure uniform thermal distribution throughout the specimen. To optimize the thermal condition and time efficiency, the subsequent specimen was co-placed in the chamber during current testing, reducing its thermal equilibration time to 5 min, thereby minimizing the total cycle time without compromising thermal stability or introducing unwanted thermal history variations. This method significantly enhances the efficiency of tensile testing at elevated temperatures.

## 3. Tensile Mechanical Responses and Analysis

Quasi-static tensile tests were conducted at strain rates of 10^−3^ s^−1^, 10^−2^ s^−1^, and 10^−1^ s^−1^, while dynamic tests were conducted at strain rates of 1 s^−1^, 20 s^−1^, and 200 s^−1^. The reported strain rates are engineering values, calculated based on the initial gauge length of the specimen and the constant crosshead speed of the testing machine. Each strain rate was tested at four temperatures: 23 °C, 50 °C, 80 °C, and 110 °C. To ensure the reliability of the experimental results, three replicate tests were conducted for each combination of loading and temperature conditions, yielding a total of 72 test specimens. Prior to testing initiation, the gauge length and critical dimensions of the specimens were measured using vernier calipers and recorded for subsequent calculation. The elongation was determined by calculating the full-field strain distribution in a non-contact manner by tracking the deformation of speckle patterns on the specimen surface using Digital Image Correlation (DIC). Engineering stress and engineering strain were adopted as the primary metrics for characterizing the macroscopic mechanical response. Engineering stress was calculated as the applied force divided by the original cross-sectional area of the specimen, and engineering strain was derived from the measured displacement normalized by the original gauge length. Engineering stress and strain were calculated using the following expressions:

(1) $$\sigma_{E}=\frac{F}{A}$$

(2) $$\varepsilon_{E}=\frac{\Delta L}{L_{0}}$$

In this work, force *F* and change in length Δ*L* are defined as signed quantities, with tension being positive and compression negative. Based on the assumption of constant volume during tensile deformation, the true stress $\sigma_{T}$ and true strain $\varepsilon_{T}$ of PEEK can be expressed as

(3) $$\sigma_{T}=\sigma_{E}\left(1+\varepsilon_{E}\right)$$

(4) $$\varepsilon_{T}=\ln\left(1+\varepsilon_{E}\right)$$

**Figure 3:**
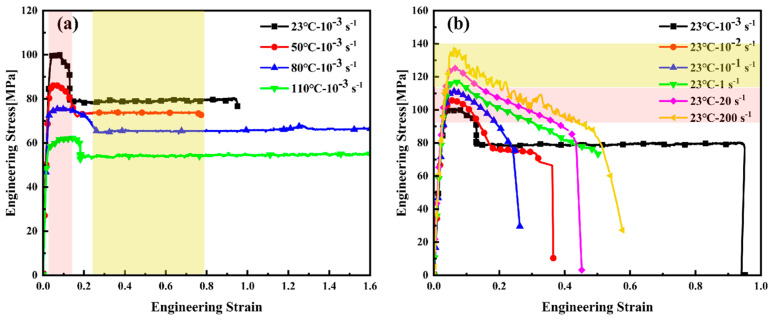
Engineering stress–strain curves of PEEK: (**a**) 10^−3^ s^−1^; (**b**) 23 °C.

Due to the satisfactory repeatability of the engineering stress–strain curves obtained under each testing condition, the curve closest to the average response was selected as the representative for each group. Taking Figure 3 as an example, Figure 3 presents the engineering stress–strain curves obtained at room temperature (23 °C) and a strain rate of 10^−3^ s^−1^. The curves exhibit characteristic viscoelastic deformation behavior, marked by an initial linear elastic region followed by nonlinear yielding and plastic flow. The observed increase in yield stress with decreasing temperature or increasing strain rate demonstrates the pronounced temperature and strain rate sensitivity of PEEK’s flow stress and yield strength. In Figure 3a, the yellow region represents the steady-state flow stress zone where the material undergoes plastic deformation, while the pink region corresponds to the yield stress platform, representing the stress level at which macroscopic yielding initiates. As shown in the figure, the steady-state flow stress decreases with increasing temperature. In Figure 3b, the pink area indicates the low-strain-rate region, where a distinct yield peak and subsequent plateau are observable, characteristic of semi-crystalline polymers deforming under quasi-static conditions. The yellow area indicates the medium-to-high-strain-rate region, where no clear yield stress plateau is observed.

**Figure 4:**
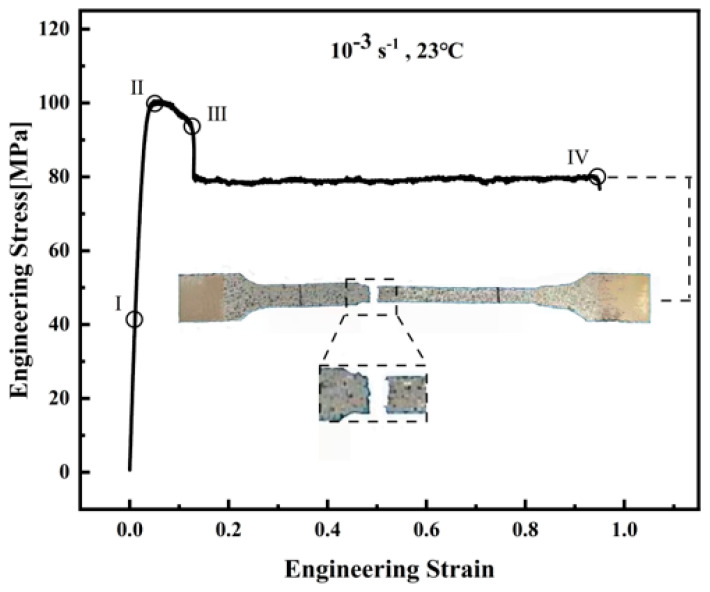
Schematic diagram of strain response for PEEK under tensile testing at 10^−3^ s^−1^ and 23 °C.

The stress–strain behavior of polyether-ether-ketone (PEEK) under uniaxial tensile loading exhibits three distinct deformation stages, which are critically influenced by its semi-crystalline microstructure. A comprehensive understanding of these stages is essential for accurate constitutive modeling and engineering applications of PEEK and PEEK-based components in demanding environments. These stages are illustrated schematically in Figure 4 and described in detail below:

1. The first stage is the pre-yield stage (elastic stage), as shown in Region I of Figure 4, where the stress–strain curve of PEEK exhibits linear elastic behavior. In this regime, the engineering stress increases proportionally with engineering strain, following Hooke’s law. The slope of this segment corresponds to the elastic modulus, a key parameter governing the material’s stiffness. The value of the elastic modulus in semi-crystalline polymers like PEEK is influenced by factors such as the crystallinity degree, molecular orientation, and testing conditions (notably temperature and strain rate).
2. When stress reaches the yield stress (strain increases without stress increasing), the second stage, the yield stage, begins. In Region II of Figure 4, PEEK demonstrates strain softening [25] after yielding, where further deformation requires lower stress than previously needed. Strain softening is a characteristic behavior of many thermoplastics, wherein the flow stress decreases after the yield peak with a further increase in strain. The extent of softening is highly sensitive to the strain rate and temperature, with higher rates and lower temperatures typically resulting in more significant softening effects.
3. The final stage is the post-yield stage (plastic stage) as shown in Region IV of Figure 4, characterized by nearly constant flow stress [26] until fracture, referred to as the steady-state flow stress. Necking phenomena can be clearly observed, wherein deformation localizes within a narrow region of the specimen, leading to a visible reduction in cross-sectional area. The neck often stabilizes and propagates along the gauge length under sustained loading, a behavior typical of ductile semi-crystalline thermoplastics. This steady plastic flow continues until eventual fracture.

**Figure 5:**
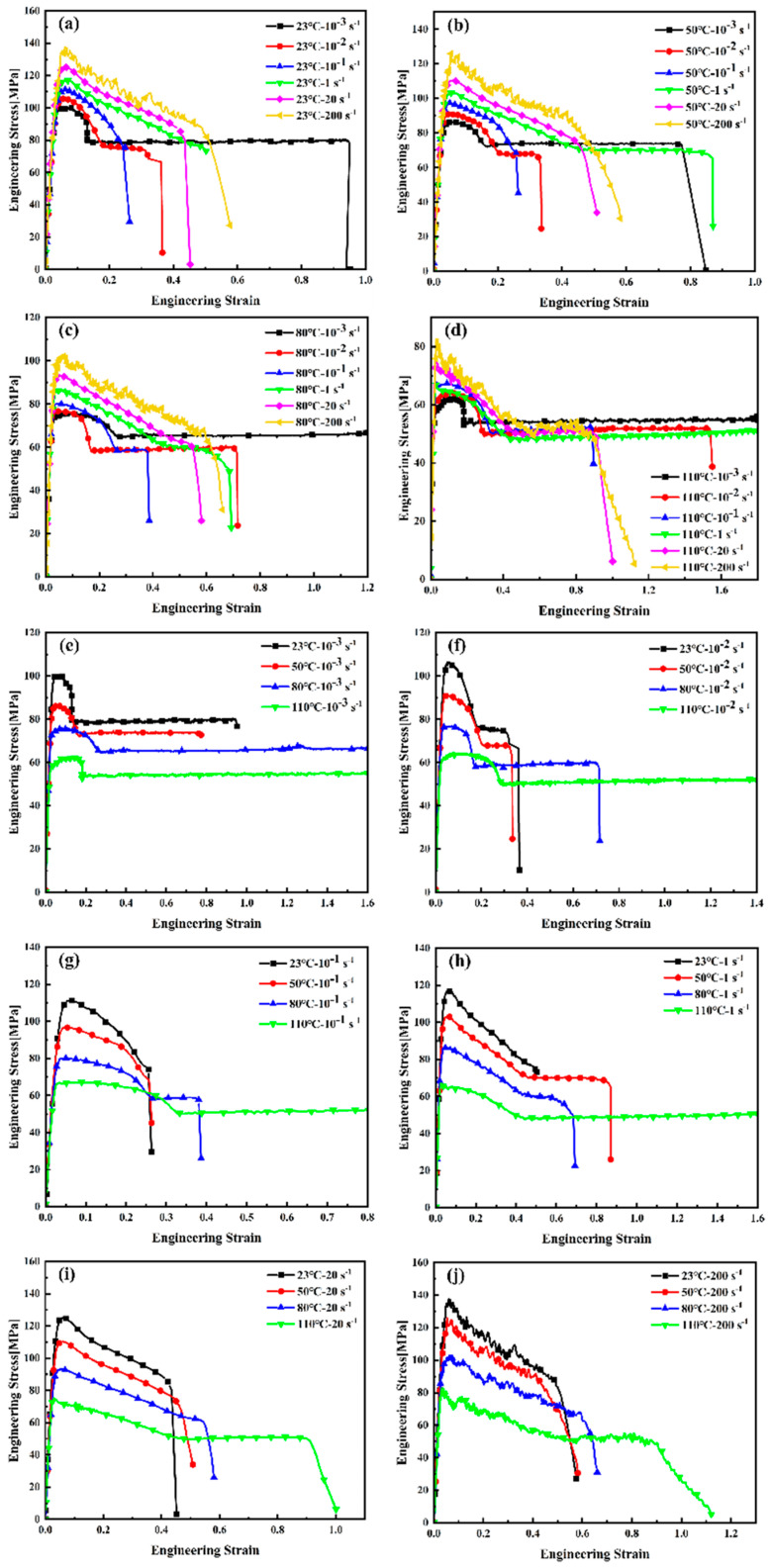
Comparison of engineering stress–strain curves of PEEK ((**a**–**d**) the same temperature; (**e**–**j**) the same strain rate).

As evidenced by the comparative analysis in Figure 5, PEEK exhibits pronounced temperature and strain rate dependence under both quasi-static and dynamic loading conditions. Since this study focuses exclusively on uniaxial stress states, the investigation primarily examines the tensile mechanical response of PEEK prior to yielding. This includes the linear elastic regime, where stress varies proportionally with strain and the slope defines the elastic modulus; the transition zone to nonlinear behavior, marking the initiation of irreversible microstructural changes; and the yield onset, where plastic deformation begins dominantly. These stages collectively form the critical foundation for understanding more complex multiaxial stress states, anisotropic responses, and post-yield phenomena such as necking, plastic instability, and fracture.

**Figure 6:**
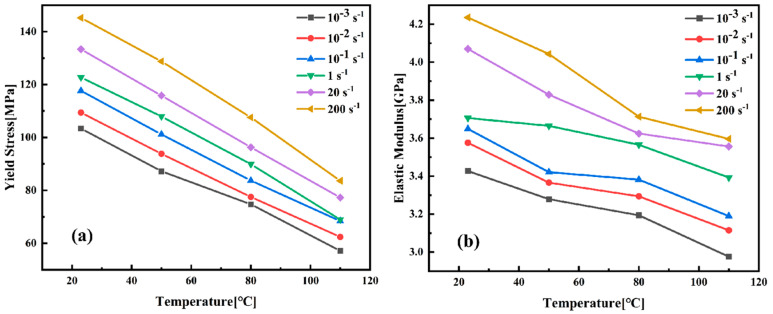
Characterization of mechanical properties of PEEK: (**a**) yield strength; (**b**) elastic modulus.

As shown in Figure 6, the elastic modulus of PEEK decreases with increasing temperature, reflecting enhanced molecular chain mobility and reduced stiffness as the material approaches its glass transition temperature (∼143 °C). As shown in Table 2 and Table 3, error bars represent the deviation range from three replicate tests. The yield stress demonstrates an approximately linear reduction, consistent with the thermal activation theory of plastic deformation in semi-crystalline polymers [27,28]. Conversely, these mechanical properties increase with increasing strain rate, though with less pronounced regularity compared to the temperature effect. The divergence in influence between thermal and rate effects underscores the necessity of employing coupled thermomechanical constitutive models. In summary, the mechanical properties of PEEK are governed by a complex interplay between temperature and strain rate, necessitating an approach to material characterization and model development for applications in demanding engineering environments.

**Figure 7:**
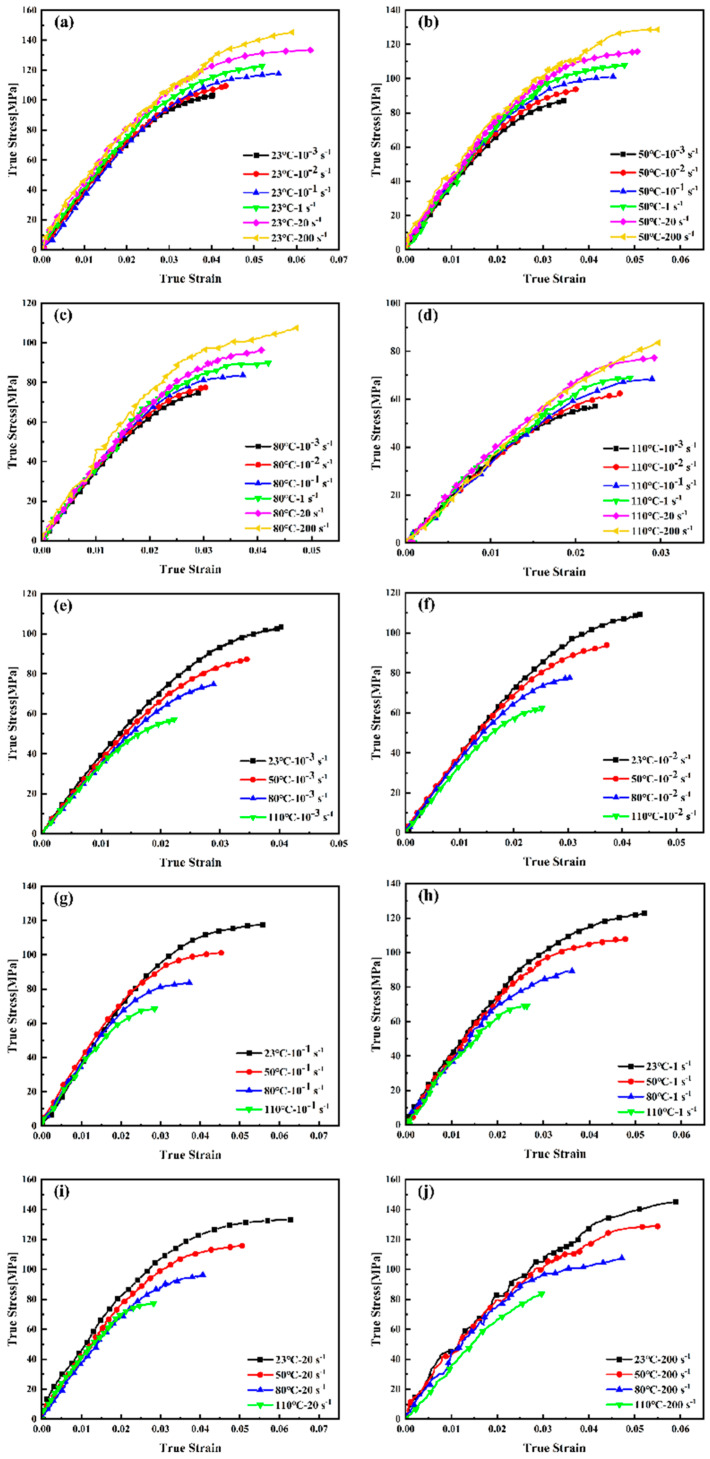
Comparison of true stress–strain curves of PEEK ((**a**–**d**) the same temperature; (**e**–**j**) the same strain rate).

The true pre-yield stress–strain curves of PEEK under varying temperature and strain rate conditions, computed using Equations (3) and (4), are presented in Figure 7.

## 4. Constitutive Model of PEEK Material

The above analysis indicates that PEEK exhibits pronounced temperature and strain rate dependence. Consequently, its constitutive behavior cannot be adequately represented by conventional mechanical constitutive equations; the equations must therefore incorporate modifications to account for the coupled effects of temperature and strain rate. Given the nonlinear viscoelastic characteristics of PEEK’s tensile mechanical response, a new phenomenological viscoelastic constitutive model is proposed based on the Sherwood–Frost framework. The Sherwood–Frost constitutive equation assumes that the effects of density, temperature, strain, and strain rate on stress can be represented by independent functions, with its general form expressed as

(5) $$\sigma =H(T)\cdot G(\rho )\cdot M(\varepsilon ,\dot{\varepsilon })\cdot f(\varepsilon )$$

where σ/MPa is the stress, T/°C is the temperature, ρ is the material density, H(T) describes the thermal softening effect, G(ρ) describes the density effect, $M(\varepsilon ,\dot{\varepsilon })$ characterizes the strain rate dependence of stress, and f(ε) is the shape function.

Based on the Seeger equation, the strain rate term can be characterized by the following Equation (6):

(6) $$M(\varepsilon ,\dot{\varepsilon })=1+C\cdot \ln\frac{\varepsilon }{\left(\dot{\varepsilon }/{\dot{\varepsilon }}_{0}\right)}$$

where $\dot{\varepsilon }$ is the strain rate, ${\dot{\varepsilon }}_{0}$ is the reference strain rate, and *C* is a fitting parameter determined through experimental data fitting. The shape function f(ε) is given by Equation (7):

(7) $$f(\varepsilon )=\sum_{i=1}^{n}A_{i}\cdot \varepsilon^{i}$$

where n and *A*_i_ are fitting parameters. Since the material used has a uniform density, density effects are not considered. In Equation (7), n = 3 was selected to achieve an optimal balance between fitting accuracy and computational efficiency. Furthermore, to reduce computational costs, the strain-rate-dependent stress function and shape function were both determined under temperature of 23 °C and strain rate of 1 s^−1^.

(8) $$\begin{array}{l} T_{0}=23\;{}^{\circ}C\\ {\dot{\varepsilon }}_{0}=1\;s^{-1} \end{array}$$

By setting H(T)=1 under these reference conditions $T_{0}$ and ${\dot{\varepsilon }}_{0}$, the constitutive relation can be expressed as follows:

(9) $$\sigma =1\cdot (1+C\cdot \ln\frac{\varepsilon }{\dot{\varepsilon }})\cdot (A_{1}\cdot \varepsilon +A_{2}\cdot \varepsilon^{2}+A_{3}\cdot \varepsilon^{3})$$

The true stress–strain curves (Figure 7) obtained from initial experimental conditions were fitted according to Equation (9); the corresponding parameters are listed in Table 4. The high goodness-of-fit, indicated by determination coefficients R^2^ exceeding 99% across all test conditions, confirms the model’s ability to accurately represent the nonlinear mechanical behavior of PEEK under both quasi-static and dynamic loading.

The temperature function H(T) was determined from experimental data obtained at 23 °C, 50 °C, 80 °C, and 110 °C and is applicable under standard strain rate condition ${\dot{\varepsilon }}_{0}$. Under this condition, any variations in the constitutive response are solely attributable to temperature effects. This selection covers a range from room temperature to approaching the glass transition temperature (Tg) of PEEK, allowing the model to account for transitions in macromolecular mobility and deformation mechanisms. The obtained values are listed in Table 5.

**Figure 8:**
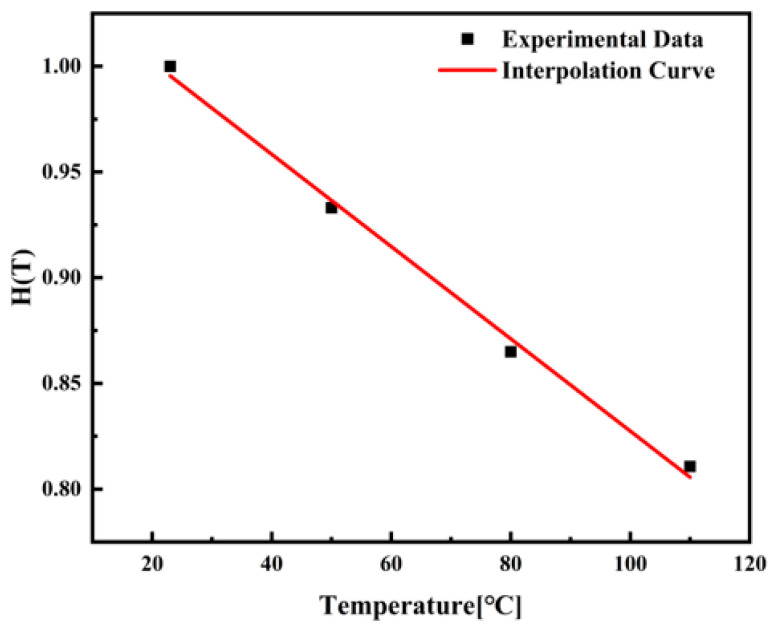
Temperature-dependent interpolation fitting curve.

Subsequently, the temperature-dependent values were fitted to derive the temperature function H(T); the fitted curve is shown in Figure 8:

(10) H(T)=1.04571−0.00218T

Substituting the above variables into Equation (9) yields the final explicit expression of the constitutive model.

**Figure 9:**
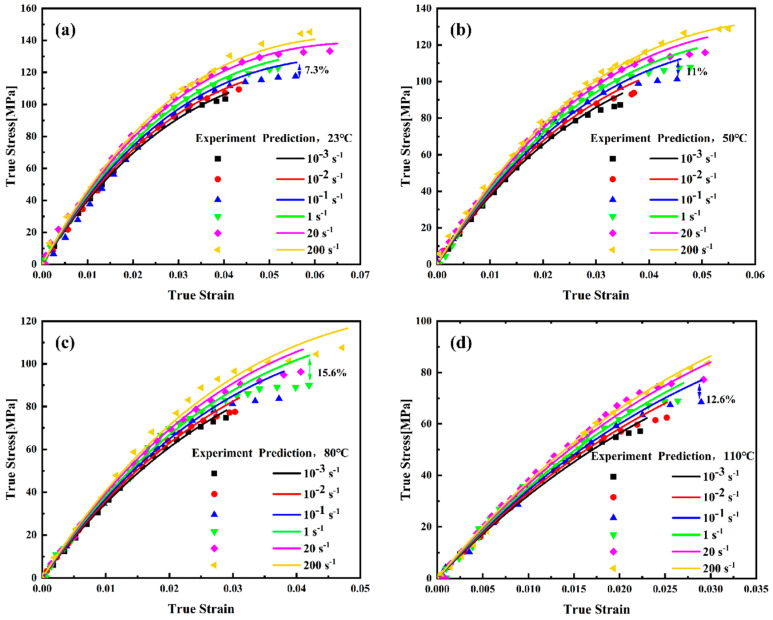
The true stress–strain curves of PEEK before yielding and model predictions: (**a**) 23 °C; (**b**) 50 °C; (**c**) 80 °C; (**d**) 110 °C.

Figure 9 presents a comparison between the fitted curves and experimental data. As shown in Figure 9, the constitutive model proposed in this study demonstrates good agreement between fitted curves and experimental data under both static and dynamic conditions. This indicates that the phenomenological nonlinear viscoelastic constitutive model can simultaneously predict the quasi-static and dynamic tensile mechanical responses of PEEK before yielding within the investigated range. Its formulation avoids undue mathematical complexity, making it well-suited for implementation in finite element simulations for engineering applications such as impact analysis, thermoforming process optimization, and performance prediction under service conditions. Despite certain deviation in accuracy, this method offers advantages in terms of computational simplicity and practical utility. The maximum error observed under most conditions remains below 10%. Furthermore, it demonstrates reliable predictive capability for the elastic modulus of PEEK across varying temperatures and strain rates. The model successfully reproduces the reduction in stiffness with increasing temperature and its increase with strain rate. Notably, the predictive capability extends to strain rates not included in the parameter identification process. For instance, the experimental data at the strain rate of 10^−1^ s^−1^ were not used for model determination, yet the maximum prediction error was 15.6%. Table 6 is a summary table comparing predicted and experimental errors (including average values and maximum values). These results demonstrate the reliability of the proposed constitutive model. 

## 5. Conclusions

This paper investigated quasi-static and dynamic tensile tests of PEEK at four temperatures ranging from 23 °C to 110 °C and six strain rates ranging from 10^−3^ s^−1^ to 200 s^−1^. The results demonstrate that the quasi-static and dynamic tensile responses of PEEK all exhibit pronounced sensitivity to both temperature and strain rate. With decreasing temperature or increasing strain rate, the yield strength, flow stress, and elastic modulus of PEEK increase.

This paper proposes a modified phenomenological nonlinear constitutive model based on the Sherwood–Frost equation to characterize the pre-yield tensile mechanical response of PEEK. This enhanced model incorporates functional terms that simultaneously account for the effects of temperature and a broad range of strain rates, effectively integrating both thermal activation theory and viscoelasticity principles. The model predictions show excellent agreement with experimental stress–strain curves, particularly in predicting the elastic modulus of PEEK in different conditions. Notably, it accurately captures the evolution of the elastic modulus with varying temperature and strain rate. Although slight deviations are observed in transition regions between elastic and viscoplastic deformation at the highest strain rates, the model maintains a maximum prediction error within 15.6%, even for data not included in parameter identification (e.g., 10^−1^ s^−1^ tests).

The results demonstrate that the constitutive model used to predict the tensile mechanical response of PEEK before yielding provides high reliability in the temperature and strain rate ranges investigated in this paper. The reliability and computational efficiency of this constitutive model make it particularly suitable for application in finite element simulations involving the design and analysis of PEEK components subjected to complex thermal–impact environments, establishing a theoretical foundation for future PEEK applications and processing techniques.

While the proposed constitutive model demonstrates agreement with experimental data within the range of low/medium strain rates (10^−3^ to 200 s^−1^), its predictive capability at strain rates significantly higher than 200 s^−1^ requires further investigation. At higher strain rates (e.g., >10^3^ s^−1^), deformation mechanisms such as pronounced adiabatic heating, changes in dominant relaxation processes, or even altered yielding behavior may become significant. These factors are not explicitly captured in the current model formulation. Therefore, extrapolation beyond the tested range should be undertaken with caution. Future work will focus on extending the experimental database to include higher strain rates to refine the model for a broader application range.

## Figures and Tables

**Table 1:** Summary of test conditions for uniaxial tensile tests.

Test Type	Test Piece Size	Testing Standard	Temperature (°C)	Strain Rate (s^−1^)	Number of Specimens
Quasi-static test	170 mm× 20 mm × 4 mm	GB/T1040	23	10^−3^, 10^−2^, 10^−1^	9
			50		9
			80		9
			110		9
Dynamic test	97 mm × 20 mm × 4 mm		23	1, 20, 200	9
			50		9
			80		9
			110		9

**Table 2:** Error bars of yield stress from triplicate tests.

Temperature Error Bars Strain Rate	10^−3^ s^−1^	10^−2^ s^−1^	10^−1^ s^−1^	1 s^−1^	20 s^−1^	200 s^−1^
23 °C	0.3–3.1%	0.9–1.2%	0.2–1.2%	0.7–1.0%	0.6–1.2%	0.5–2.3%
50 °C	0.3–1.1%	0.1–2.7%	1.9–2.4%	1.4–2.8%	0.9–1.3%	0.3–0.7%
80 °C	1.4–2.5%	0.4–0.5%	0.1–0.3%	0.7–1.4%	0.7–1.5%	1.1–1.2%
110 °C	0.2–1.1%	0.9–1.4%	0.9–1.0%	1.1–1.5%	1.9–5.7%	0.4–1.8%

**Table 3:** Error bars of elastic modulus from triplicate tests.

Temperature Error Bars Strain Rate	10^−3^ s^−1^	10^−2^ s^−1^	10^−1^ s^−1^	1 s^−1^	20 s^−1^	200 s^−1^
23 °C	0.1–1.4%	1.4–2.4%	0.3–1.5%	1.7–1.9%	0.4–6.5%	1.1–2.2%
50 °C	0.7–1.0%	0.4–2.1%	0.4–0.6%	0.8–6.1%	4.2–5.6%	1.1–1.3%
80 °C	0.3–3.3%	0.1–0.9%	0.2–1.3%	0.4–2.1%	2.9–4.4%	1.2–2.5%
110 °C	0.3–2.3%	2.2–3.6%	0.2–2.0%	1.4–4.5%	0.8–7.0%	2.1–5.9%

**Table 4:** Parameters A_n_ for the shape function f(ε).

*T*/°C	*C*	*A*_1_	*A*_2_	*A*_3_	*R*^2^/%
23	−0.01329	4648.016	−54,460.575	207,019.487	99.81

**Table 5:** The values of temperature-dependent function H(T).

*T*/°C	*H*(*T*)	*R*^2^/%
23	1	-
50	0.93316	99.76
80	0.86501	99.66
110	0.81074	99.41

**Table 6:** Summary table comparing predicted and experimental errors.

Condition	Average Value	Maximum Value
23 °C	4.3%	7.3%
50 °C	7.3%	11.0%
80 °C	10.6%	15.6%
110 °C	8.9%	12.6%
10^−3^ s^−1^	6.2%	8.9%
10^−2^ s^−1^	7.3%	9.5%
10^−1^ s^−1^	11.6%	15.3%
1 s^−1^	9.8%	15.6%
20 s^−1^	7.6%	10.9%
200 s^−1^	4.1%	8.4%
Overall	8.9%	15.6%

## Data Availability

The original contributions presented in this study are included in the article. Further inquiries can be directed to the corresponding authors.
